# Editorial: Dysregulated Protein Homeostasis in the Aging Organism

**DOI:** 10.3389/fmolb.2021.788118

**Published:** 2021-10-14

**Authors:** Thorsten Pfirrmann, Niki Chondrogianni, Heidi Olzscha, Aphrodite Vasilaki

**Affiliations:** ^1^ Department of Medicine, Health and Medical University, Potsdam, Germany; ^2^ Institute of Physiological Chemistry, Martin-Luther University, Halle, Germany; ^3^ Institute of Chemical Biology, National Hellenic Research Foundation, Athens, Greece; ^4^ Medical School Hamburg, Medical Faculty, Institute of Molecular Medicine, Hamburg, Germany; ^5^ Institute of Life Course and Medical Sciences, University of Liverpool, Liverpool, United Kingdom

**Keywords:** proteostasis, protein folding, ubiquitin proteasome system, autophagy, organismal aging, protein modification

**FIGURE 1:**
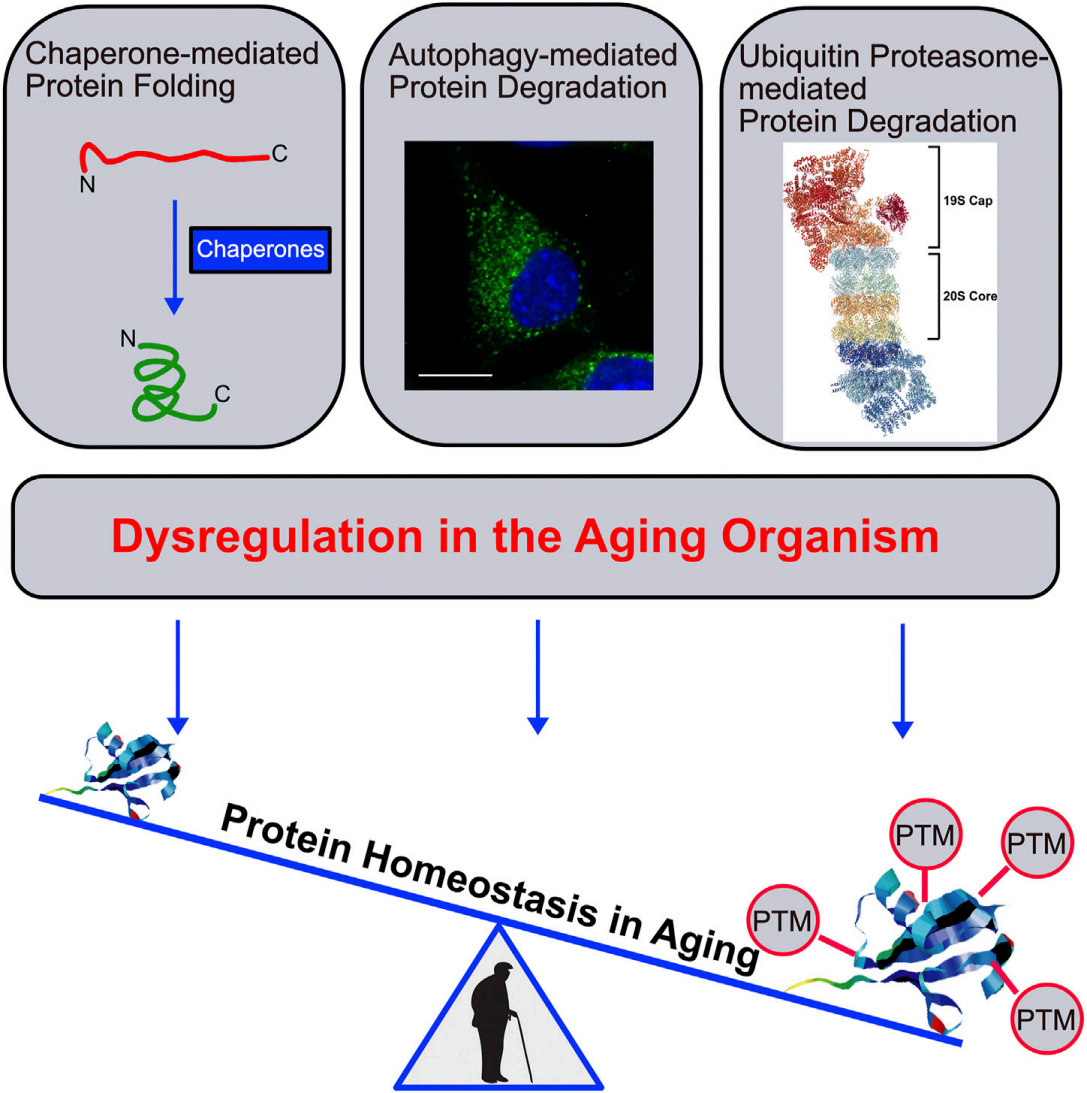
Dysregulated protein homeostasis in the aging organism.

Cellular protein homeostasis is defined as the fine-tuned balance between protein synthesis, folding and degradation at the level of functional proteins. In healthy cells and organisms, this balance is carefully maintained through regulatory quality control systems, including protein folding mechanisms and protein degradation through the ubiquitin proteasome system (UPS), and the autophagy-lysosome system (ALS) (Balch et al., 2008). A prominent hallmark of aging is the accumulation of damaged and misfolded proteins that are prone to aggregation. In other words, dysregulated protein homeostasis correlates with time and is considered as one of the major drivers of the aging process. Dysregulated protein homeostasis arises from dysfunctional chaperone-mediated protein folding and/or impaired UPS- and/or ALS-mediated protein degradation and is associated with several age-related diseases including Alzheimer’s and Parkinson’s disease, among many others (Figure 1) (López-Otín et al., 2013). The accumulation of posttranslational modified proteins that accumulate over time is another striking feature in aging organisms (Baldensperger et al., 2020). On the one hand, this accumulation is partly a consequence of the failing protein homeostasis, on the other hand it actively contributes to the dysfunction of protein refolding and degradation as demonstrated in the case of protein glycation (Uchiki et al., 2012) and oxidation (Breusing and Grune, 2008). Interestingly, in several instances reversal or delayed accumulation of some of these protein modifications reverses or slows down aging phenotypes (Asif et al., 2000). A better understanding of such processes is not only of scientific interest but will have a strong impact on the progression of therapies against age-related diseases.

This Research Topic is a compilation of one Review, three Mini Reviews and an Original Research article and combines recent progress in the field of protein homeostasis. A distinct focus of this research topic is directed towards enzymatic and non-enzymatic protein modifications that accumulate over time and are associated with dysregulated protein homeostasis. Proteins destined for UPS-mediated degradation require “the kiss of death”, the attachment of a polyubiquitin chain onto the target protein (Komander and Rape, 2012). During aging, several cells begin to express a frameshift-mutated ubiquitin called UBB^+1^, that can disrupt proteasome function but simultaneously improves stress resistance and extends lifespan at low concentrations. Banasiak et al. review the molecular basis of how UBB^+1^ affects UPS performance along with its dose-dependent action as a cytoprotective or cytotoxic molecule.

Effective protein polyubiquitination requires the formation of an isopeptide bond between the C-terminal glycine residue of ubiquitin and the amino group of a lysine residue either within the substrate or within ubiquitin to form K48-linked polyubiquitinated substrates. This modification is the most prominent inducer of substrate unfolding and degradation by the proteasome. Recent evidence highlights non-enzymatic lysine acylation reactions by reactive acyl-CoA species, acyl phosphates, and α-dicarbonyls. Baldensperger et al. review the potential of lysine acylation to interfere with lysine ubiquitination as a possible molecular mechanism of dysregulated protein homeostasis in the aging organism and age-related diseases. Furthermore, the review elegantly summarizes the metabolic source of such intermediates and the reaction mechanisms of their formation to give an overview of the resulting acyl lysine modifications and their downstream effects.

In a pioneering study Franzka et al. provide a comprehensive MALDI-TOF based analysis of the cardiac glycoproteome of mice at different ages and show that the murine cardiac glycoproteome changes during aging and includes increasing incorporation of mannose residues into carbohydrate chains. Accordingly, the authors reveal an age-related increase of GDP-mannose pyrophosphorylase B (GMPPB), the enzyme that facilitates the supply of the sugar-donor GDP-mannose. Different approaches to isolate glycosylated proteins from young and old mice reveals quantitative changes in the cardiac glycoproteome including proteins involved in the formation of the extracellular matrix and Ca^2+^-binding proteins of the endoplasmic reticulum. The authors propose that changes in the heart glycoproteome likely contribute to age-related functional decline of the cardiovascular system.

As the key transcription factor of the renin-angiotensin-aldosterone-system, the mineralocorticoid receptor (MR) mediates electrolyte and blood pressure homeostasis and consequently contributes to pathologies of the cardiovascular system. During aging, the activity of MR increases independent of its ligand aldosterone and contributes to the progression of cardiovascular diseases. In a comprehensive Review Article, Gadasheva et al. summarize different posttranslational modifications of MR that contribute to the aberrant MR activation in the aging organism. The authors systematically summarize posttranslational modifications of MR that are either enzymatically catalyzed or of non-enzymatic nature and describe their impact on MR receptors stability, ligand-binding, transformation, co-regulator binding, DNA-binding and transactivation.

Ruano reviews the molecular alterations occurring during normal aging in the most relevant protein quality control systems such as the molecular chaperones, the UPS, and the ALS. Additionally, the authors comprehensively review how these systems are entangled with each other and functionally cooperate. Interestingly, the authors summarize the role of inflammation, as a synergistic negative factor of the protein quality control systems during normal aging.

In conclusion, this research topic gathered five articles addressing novel and updated aspects related to protein homeostasis in the aging organism, covering enzymatic and non-enzymatic posttranslational modifications that accumulate in aging organisms and likely trigger the aging process or contribute to the manifestation of age-related diseases.
